# The thioredoxin‐like and one glutaredoxin domain are required to rescue the iron‐starvation phenotype of HeLa *GLRX3* knock out cells

**DOI:** 10.1002/1873-3468.70072

**Published:** 2025-05-21

**Authors:** Laura Magdalena Jordt, Manuela Gellert, Finja Zelms, Sander Bekeschus, Christopher Horst Lillig

**Affiliations:** ^1^ The Institute for Medical Biochemistry and Molecular Biology University Medicine Greifswald, University of Greifswald Germany; ^2^ Clinic and Polyclinic for Dermatology and Venereology University Medicine Rostock Germany; ^3^ The Leibniz Institute for Plasma Science and Technology e.V. (INP) Greifswald Germany

**Keywords:** class I glutaredoxin, glutaredoxin 3, iron homeostasis, iron metabolism, thioredoxin

## Abstract

Glutaredoxin 3 (Grx3) is a multidomain protein (Trx‐GrxA‐GrxB) with a Trx‐like domain and two Grx domains containing a CGFS motif for binding Fe2S2 clusters. To study the function of these domains, HeLa cells with GLRX3 knockout were generated via CRISPR/Cas. The knockout activated iron‐regulatory protein 1, indicating iron starvation due to impaired iron metabolism. Transfection with constructs encoding wild‐type or individual domains showed that only the Trx‐GrxA construct could rescue the phenotype, matching the effect of full‐length Grx3. The specific role of the second Grx domain in human Grx3, absent in simpler eukaryotes such as yeast, remains unclear. While the individual domains are insufficient to rescue the knockout of full‐length Grx3, the Trx‐GrxA module is functionally critical.

**Impact statement**

Glutaredoxin 3 (Grx3) contains a Trx‐like domain and two Grx domains. The importance of the domains in higher eukaryotes has not previously been addressed in physiological or cellular contexts. Here, we report GLRX3 knockout results in activation of iron regulatory protein 1, and a Trx‐GrxA construct could rescue the phenotype.

## Graphical abstract

Glutaredoxin (Grx) 3 proteins contain a thioredoxin domain and one to three class II Grx domains. These proteins play a crucial role in iron homeostasis in eukaryotic cells. In human Grx3, at least one of the two Grx domains, together with the thioredoxin domain, is essential for its function in iron metabolism.

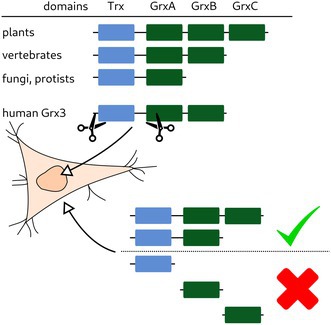

## Abbreviations

**CIA**, cytosolic iron–sulfur cluster assembly

**FeS**, iron–sulfur

**ft**, ferritin

**GPAT**, amidophosphoribosyl transferase

**Grx**, glutaredoxin

**IRE**, iron‐responsive element

**IRP**, iron‐regulatory protein

**PKC**, protein kinase C

**TfR**, transferrin receptor

**Trx**, thioredoxin

The maturation of cytosolic iron–sulfur (FeS) proteins depends on a low‐molecular‐weight component provided by the mitochondrial synthesis machinery, the primary place for FeS synthesis. This yet‐to‐be‐defined component is received by the cytosolic iron–sulfur cluster assembly (CIA) machinery. This machinery functions to assemble and deliver FeS clusters to target proteins; for overviews, see [1, 2, 3]. Glutaredoxin 3 (Grx3), together with the BolA‐like protein BolA2, is a candidate for the trafficking and delivery of FeS clusters to the CIA machinery [4].

Human Grx3, encoded by the *GLRX3* gene and also known as PICOT, TXNL‐2, or HUSSY‐22, is a member of the thioredoxin (Trx) protein family. The cytosolic and nuclear protein is ubiquitously expressed and belongs to the multidomain class II glutaredoxins [5]. The protein consists of an N‐terminal Trx‐like domain and two C‐terminal Grx domains containing the characteristic Cys‐Gly‐Phe‐Ser active site motif. This motif is required to complex iron–sulfur (FeS) clusters [6]. Like other FeS‐Grxs, Grx3 can form a homodimeric complex bridged by Fe_2_S_2_‐clusters. These clusters are ligated by the active site cysteinyl thiols of the two Grx domains and by the sulfhydryl groups of two glutathione molecules, noncovalently bound to each Grx domain [6].

Initially, Grx3 was described as an interaction partner of protein kinase C theta (PKC); it was implicated in several signaling pathways that lead to the activation of cells, *that is*, immune cell response, cell cycle progression during embryogenesis, cancer cell growth, and metastasis [7, 8, 9, 10]. Furthermore, Grx3 was found to be a negative regulator of cardiac hypertrophy. In animal models of cardiac hypertrophy, Grx3 was upregulated, and heterozygous Grx3^+/−^ mice were more vulnerable to developing cardiac hypertrophy [11, 12, 13]. Homozygous inactivation of the *GLRX3* gene in mice leads to embryonic death between E12.5 and E14.5; although no defects during organogenesis could be observed, the embryos developed hemorrhages in the head at E12.5 [13]. This time point also marks the start of erythropoiesis in the fetal liver, which heavily depends on heme synthesis and iron homeostasis [14]. Silencing of Grx3 in zebrafish impaired heme biosynthesis and erythropoiesis [15]; silencing of Grx3 in HeLa cells impaired iron homeostasis and caused defects in the maturation of cytosolic Fe/S‐proteins [15]. Specifically, the levels of the iron storage protein ferritin (Ft) were decreased, and the levels of the transferrin receptor (TfR) were increased. The levels of these proteins are controlled posttranscriptionally by iron‐regulatory proteins (IRPs). IRPs bind to iron‐responsive elements (IREs), hairpin structures in the untranslated region of specific mRNAs. This binding prevents, *for example*, the translation of Ft and stabilizes the mRNA encoding the TfR [16, 17, 18]. IRP1 is, in its Fe_4_S_4_‐cluster form, active as cytosolic aconitase. Loss of this cluster activates the protein as IRP. In the absence of Grx3, cytosolic aconitase activity decreased [15]. Silencing of Grx3 expression increased the binding of IRP1 to the labeled IRE of human ferritin mRNA but not IRP2. Characteristic of defects in FeS biosynthesis, Grx3‐depleted cells displayed decreased levels of Ft, increased levels of TfR, and decreased levels of the FeS‐protein amidophosphoribosyl transferase (GPAT) [15]. The latter is indicative of insufficient FeS insertion and, thus, destabilization of the protein [19, 20]. Altogether, these effects resemble a functional iron starvation phenotype.

The *Saccharomyces cerevisiae* genome encodes two homologs of Grx3 named Grx3 and Grx4. In contrast to their human counterpart, these consist of the N‐terminal Trx‐like domain and only one class II Grx domain. The proteins complex one Fe_2_S_2_‐cluster in a dimeric holo complex [21]. Yeast Grx3/4 function in intracellular iron trafficking and sensing. Depletion of Grx3/4 specifically impairs all iron‐requiring reactions in the cell, including FeS cluster biogenesis, heme synthesis, and the maturation of the di‐iron center in ribonucleotide reductase [21]. Plant genomes also encode one cytosolic/nuclear multidomain Grx, *for example*, GrxS17 in *Arabidopsis thaliana* reviewed in [22]. These proteins contain three class II Grx domains following the N‐terminal Trx‐like domain. Their function, however, appears to be more comprehensive; the protein was, for instance, implied in heat tolerance and auxin signaling [23]. Similar to other eukaryotes, GrxS17 associates with components of the cytosolic FeS cluster assembly machinery and contributes to FeS maturation, but in contrast to yeast and mammals, the protein is not essential in plants [24].

The importance of the individual domains and, moreover, the additional class II Grxs domains in higher eukaryotic organisms has not been addressed in a physiological or cellular context. In this study, we generated HeLa Grx3^−/−^‐cells using the CRISPR/Cas technique and used these cells to study the rescue of the iron‐starvation phenotype by the individual Grx3 domains and combinations thereof.

## Materials and methods

### Cell culture

Cells were cultivated at 37 °C with 5% CO_2_ in saturated atmospheric humidity in low glucose (1 g·L^−1^) DMEM supplemented with 100 U·mL^−1^ penicillin, 0.1 mg·mL^−1^ streptomycin, and 10% FCS (PAN‐Biotech, Aidenbach, Germany). At 90% confluence, cells were passaged every 3 to 4 days with a ratio of 1:10 (HeLa wt) and 1:5 (HeLa *GLRX3* ^−/−^ lines).

The parental HeLa cell line is regularly authenticated (at least every 3 years) by analysis of both phenotype (morphology and motility) and karyotype. The cell lines used in this study were confirmed to be free of Mycoplama sp. contamination using PCR.

### Transfection and generation of HeLa GLRX3^−/−^ cell lines

HeLa cells were transfected by electroporation. 3.5·10^6^ cells were resuspended in 550 μL electroporation buffer (21 mmol·L^−1^ HEPES, 137 mmol·L^−1^ NaCl, 5 mmol·L^−1^ KCl, 0.7 mmol·L^−1^ Na_2_HPO_4_, 6 mmol·L^−1^ d‐glucose, pH 7.15), mixed with 15 μg plasmid and electroporated at 250 V, 1500 μF, and 500 Ω using the BTX ECM 830. 550 μL FCS was immediately added to the cells before seeding them in 1:5 conditioned medium. The medium was changed 24 h after transfection, and cells were harvested 24–48 h after transfection. In the case of CRISPR/Cas9 plasmids (HS0000193706, HS0000193707, Sigma Aldrich/ Merck, St. Louis, MI, USA), cells were transfected with 15 μg each. Single‐cell sorting into 96‐well plates was performed via fluorescence‐activated cell sorting 24 h after transfection. Single positive transfected cells (Cas9‐coupled GFP signal) were sorted into individual cavities of a 96‐well plate containing culture medium with 20% FCS. 24 h later, the medium was changed carefully, and the sorting was assessed by fluorescence microscopy. Stable isogenic cell lines were grown from single cells. Successful knockout was confirmed by western blot, PCR, and sequencing. The primer pairs for control PCR were designed to bind the intron region before and after, and also within the excised exon 4 (intron 3 forward 5′‐CTGTTGTCCCCTTTAAAAATCTGC‐3′; exon 4 forward 5′‐CATCTTAAAGAAGATCTCAACCTTCG‐3′; exon 4 reverse 5′‐CGAAGGTTGAGATCTTCTTTAAGATG‐3′ intron 5 reverse 5′‐CTGAGACCCCTTTTGGTTCC‐3′).

### Cell lysis and fractionation

Cells were harvested by trypsinization, centrifuged, and washed once with PBS before 20‐min lysis (150 mmol·L^−1^ NaCl, 50 mmol·L^−1^ Tris, 1% (w/v) CHAPS, pH7.5 including complete protease inhibitor (Roche, Basel, Switzerland), at room temperature. Samples were stored at −80 °C. For fractionation [25], the cells were harvested as described above. The PBS‐washed cell pellet was resuspended in ice‐cold fractionation buffer (5 mmol·L^−1^ Tris/HCl, 250 mmol·L^−1^ sucrose, 1 mmol·L^−1^ EDTA, 1 mmol·L^−1^ EGTA, 1.5 mmol·L^−1^ MgCl_2_, 1 mmol·L^−1^ PMSF, pH 7.4) containing 0.008% Digitonin, with a maximum protein concentration of 2 mg·mL^−1^, and incubated for 10 min on ice. 200 μL of this suspension was taken as “whole cell lysate,” the remaining suspension was centrifuged at 14 000 ***g*** for 10 min at 4 °C. The supernatant was collected as a cytosolic fraction, and the pellet resuspended in 250 μL fractionation buffer as a mitochondria‐enriched fraction. All fractions were snap‐frozen in liquid nitrogen.

### SDS/PAGE and western blotting

Cell lysates were thawed on ice and centrifuged at 4 °C for 15 min at 17000 ***g***. The protein content of the supernatants was determined by the Bradford method (BioRad, Hercules, CA, USA). 20–40 μg of total protein was diluted in TE‐buffer (10 mmol·L^−1^ Tris, 1 mmol·L^−1^ EDTA, pH 8) and fivefold sample buffer (0.3 m Tris/HCl pH7, 50% glycerol, 5% SDS, 1 mmol·L^−1^ EDTA, 0.1% bromphenol blue). Samples were reduced with 50 mmol·L^−1^ DTT at room temperature for 30 min. Proteins were separated by SDS/PAGE using Mini‐Protean TGX stain‐free 4–20% precast gels (BioRad). The protein content of all gels (and later membranes) was imaged after activation using BioRads stain‐free technology with the Chemidoc XRS+ System. Subsequent western blotting was performed according to the manufacturer's protocol using the Trans‐Blot Turbo RTA Transfer Kit with PVDF membranes (BioRad). Membranes were blocked for 1 h at room temperature with 5% nonfat milk powder and 1% BSA in Tris‐buffered saline containing 0.05% Tween20 and incubated with specific primary antibodies against human or mouse Grx3 [26], BolA2 (Santa Cruz sc‐163 747), ferritin heavy chain (Abcam ab75973, recombinant monoclonal antibody), GPAT (kindly supplied by R. Lill, Marburg, Germany), transferrin receptor (Life Technologies, 13‐6890; Carlsbad, CA, USA), Ciapin1 (Abcam, ab154904; Cambridge, UK), or Ciao3 (Antibodies online, ABIN653781) at 4 °C overnight. Antigen–antibody complexes were detected by the Chemidoc XRS+ system using horseradish‐coupled secondary antibodies (BioRad) and enhanced chemiluminescence. Densiometric analysis was performed using ImageJ and the ImageLab 5.0 software (BioRad).

### Enzyme activity assays

Cell fractions generated as described above were subjected to colorimetric enzyme activity assays to determine the activity of aconitase, lactate dehydrogenase, and citrate synthase [27]. Samples were centrifuged 10 min at 4 °C and 10 000· ***g*** before use. Aconitase activity was measured at 340 nm in 2.5‐min intervals for 1 h using 10 μL of cytosolic or mitochondrial fractions (in a mixture of 100 mmol·L^−1^ triethanolamine, 1.5 mmol·L^−1^ MgCl_2_, 0.1 Triton X‐100, pH 8.0; 5 mmol·L^−1^ NADP^+^, 800 μm cis‐aconitate, 1.6 mU isocitrate dehydrogenase in 250 μL total volume per well). Lactate dehydrogenase activity was measured at 340 nm in 2.5 min intervals for 30 min using 10 μL of cytosolic or mitochondrial fractions (in a mixture of 50 mmol·L^−1^ Tris, 1 mmol·L^−1^ EDTA, 0.1% Triton X‐100, pH 7.4; 3 mmol·L^−1^ sodium pyruvate, 200 μmol·L^−1^ in a total volume of 250 μL per well). Citrate synthase activity was measured at 412 nm in 2.5‐min intervals for 30 min using 10 μL of cytosolic or mitochondrial fractions (in a mixture of 50 mmol·L^−1^ Tris, 50 mmol·L^−1^ NaCl, 0.5 mmol·L^−1^ DTNB, 0.1% Triton X‐100, pH 8.0; 0.1 mg·mL^−1^ acetyl‐CoA, 0.1 mg·mL^−1^ oxalacetate in 250 μL total volume per well).

### Statistical analyses

Data were subjected to unpaired two‐tailed *t‐test* comparisons to the respective controls using GraphPad.

### Cloning of Grx3 domains in pExpress

Human Grx3 and a variation of human Grx3 domains were cloned into the pExpress plasmid [28] for expression in eukaryotic cells. All constructs were amplified by PCR from a human Grx3‐pET15b plasmid [6] using the specific oligonucleotides listed in Table 1, digested with NheI and BamHI, and cloned into the NheI/BglII restricted pExpress vector. Site‐directed mutagenesis was performed by rolling circle PCR using the indicated primer pair (Table 1).

**Table 1:** Oligonucleotides used for the cloning of the different human Grx3 constructs (5′ to 3′).

Protein	Oligonucleotides
Grx3 wt	Fwd: CACACAGCTAGCATGGCGGCGGGGGCGGCTG Rev: CACACAGGATCCTTAATTTTCTCCTCTCAGTATAGGCAGCAATTCACCATTTTCTTTCAGTTCCTTCACAATATCCAATCCTCC
Grx3 ΔgrxA,B	Fwd: CACACAGCTAGCATGGCGGCGGGGGCGGCTG Rev: GGATCCTCAGGAGCCACTAGATGCATGTCGCTG
Grx3 ΔgrxB	Fwd: CACACAGCTAGCATGGCGGCGGGGGCGGCTG Rev: CACACAGGATCCTCATAATTTGGGAGCTTTGGGACAAATTGTATC
Grx3 Δtrx,grxB	Fwd: CACACAGCTAGCATGGCTAATGAACATCTTAAAGAAGATCTCAACC Rev: CACACAGGATCCTCATAATTTGGGAGCTTTGGGACAAATTGTATC
Grx3 Δtrx,grxA	Fwd: CACACAGCTAGCATGTTAGAGGAAAGGCTCAAAGTGCTGAC Rev: CACACAGGATCCTTAATTTTCTCCTCTCAGTATAGGCAGCAATTCACCATTTTCTTTCAGTTCCTTCACAATATCCAATCCTCC
C159S mutation	Fwd: CTCAAGAACCACGCTCTGGTTTCAGCAAGC Rev: GCTTGCTGAAACCAGAGCGTGGTTCTTGAG

### Sequencing

The successful generation of the homozygous knockout cell lines (oligonucleotide: CTGTTGGTCCCCTTTAAAAATCTGC) and the expression constructs (oligonucleotides: TGCTGGTTATTGTGCTGTCTC and GTGGTATGGCTGATTATG) was confirmed by DNA sequencing using services provided by Seqlab (Göttingen, Germany).

## Results

### Generation and validation of the *GLRX3* knockout cell lines

**Fig. 1:**
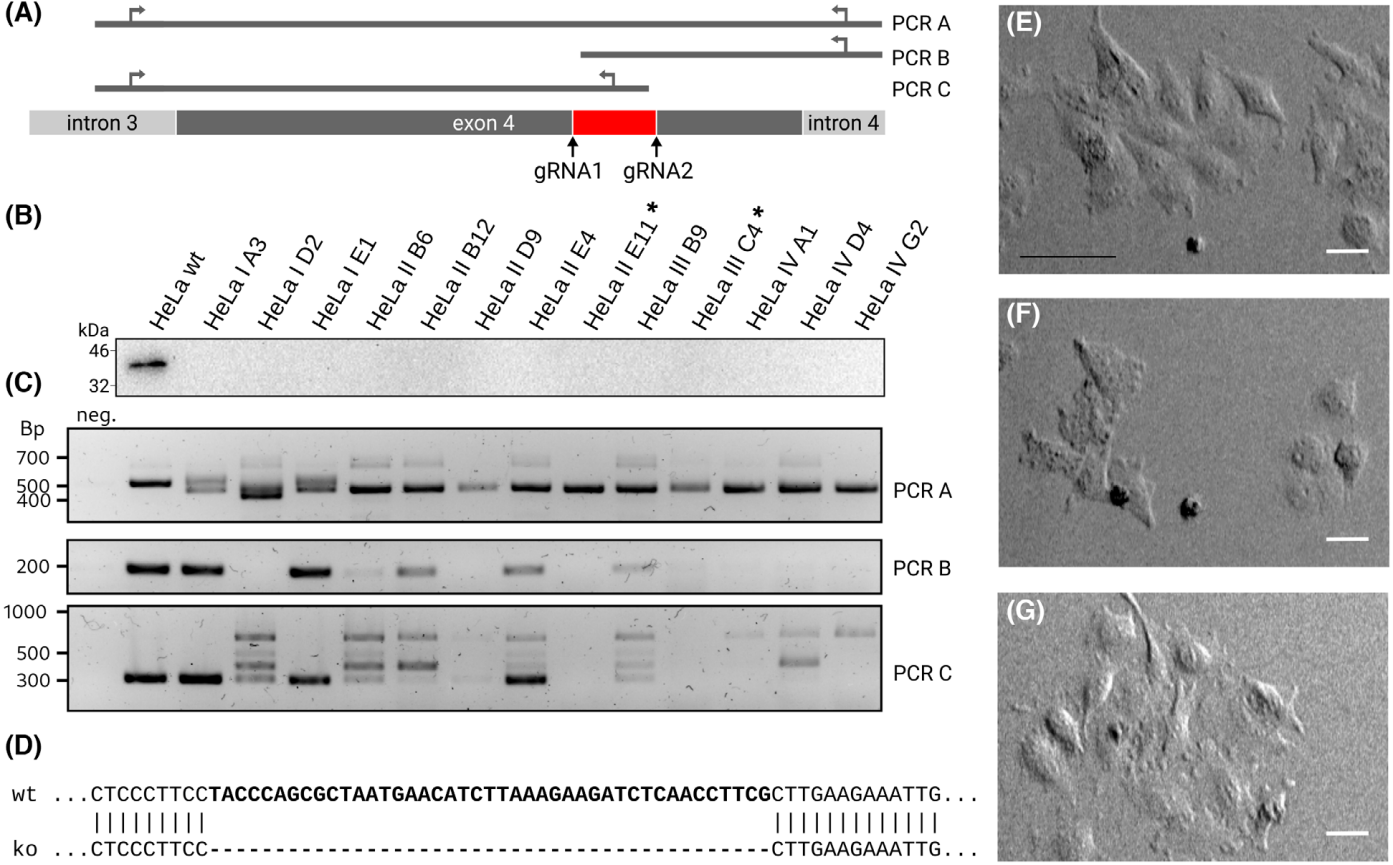
Establishing the GLRX^−/−^‐cell lines. (A) Target area for the two guide RNAs (gRNAs) used for the CrispR/Cas‐mediated knock‐out and design of the three control PCRs. (B) Western blot of candidate cell lines stained with the human Grx3‐specific polyclonal antibody. (C) Control PCRs 1–3, see A, for the candidate cell lines. Two cell lines, marked with an asterisk (*) showed a complete homozygous deletion of the target region. (D) Confirmation of the deletion in the two chosen cell lines by sequencing (identical for both cell lines). (E–G) Phase contrast microscopy of the cell lines HeLa Grx3^−/−^‐2‐E11 (E), HeLa Grx3^−/−^‐3‐C4 (F), and HeLa wild‐type. The scale bar in (E–G) indicates 10 μm.

Based on the CRISPR/Cas9 technique, a *GLRX3* knockout cell line was generated (Fig. 1A). Two guide RNAs within exon 4 of the *GLRX3* gene were transfected; isogenic cell lines were established from FACS‐sorted single cells. The loss of Grx3 was confirmed in 13 of these cell lines on the protein level using western blotting (Fig. 1B). To examine the successful *GLRX3* knockout on the genomic level, independent PCRs were established amplifying (a) the entire region from intron 3 to 5, and (b and c) fragments within exon 4. After successfully removing the target section, reaction products would be truncated by 43 bp. PCR b and c would not yield products. One primer for each of these reactions was located within the deleted target section. On the genomic level, we identified two cells in which the entire target sequence was deleted homozygously, HeLa‐2‐E11‐*GLRX3* ^−/−^ and HeLa‐3‐C4‐*GLRX3* ^−/−^ (Fig. 1C). These two clones were further analyzed by sequencing; both contained the expected 43 bp deletion of the target section in exon 4 (Fig. 1D). The deletion is located at the end of the region that encodes the Trx domain and includes a non‐sense mutation. Both cell lines were viable, and their morphology did not differ from that of the parental HeLa cells (Fig. 1E–G). However, the generation time of both cell lines was 2 days, compared to one day for the wild‐type cells.

### Characterization of the *GLRX3* knock‐out cell lines

**Fig. 2:**
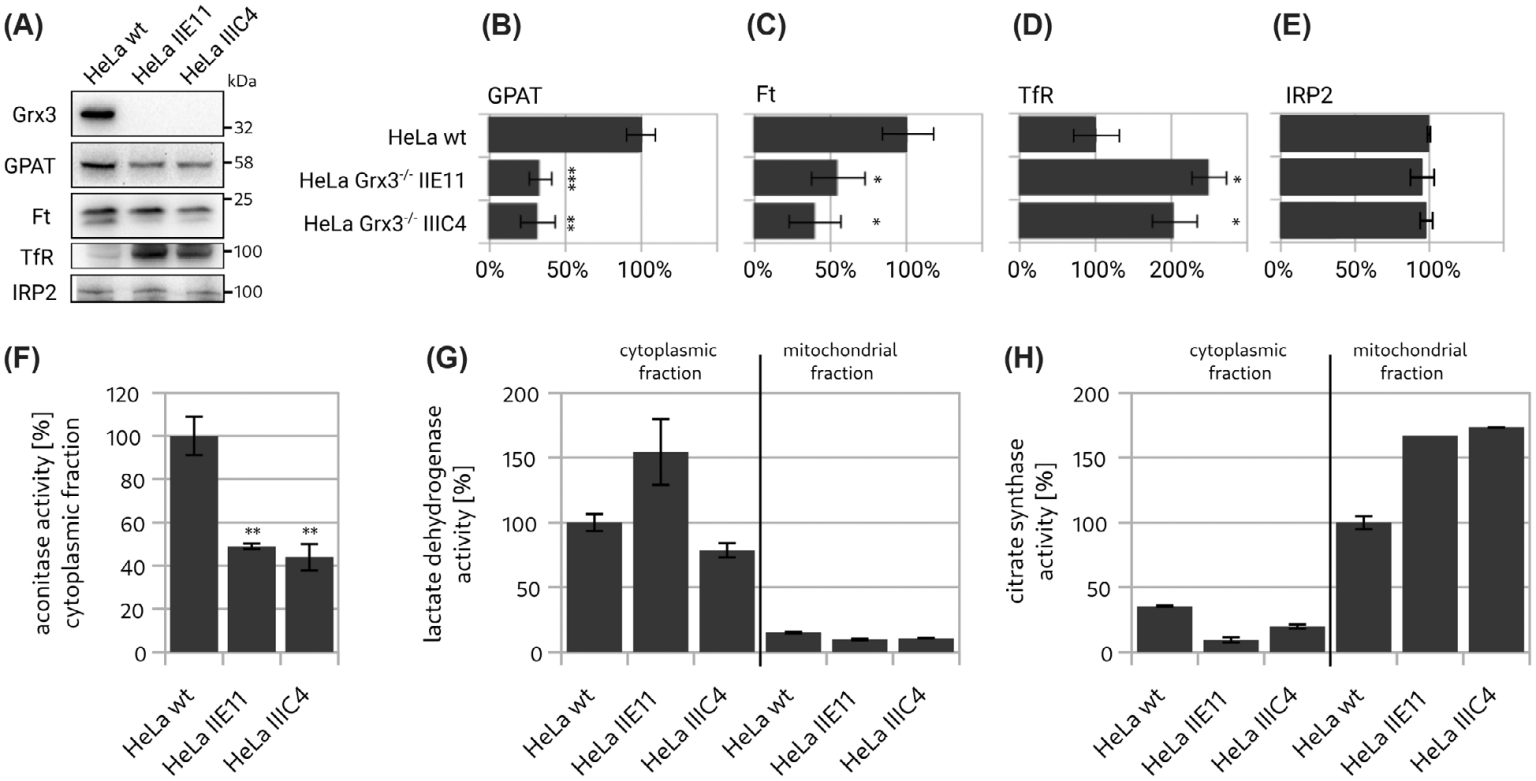
Iron‐starvation phenotype of the Grx3^−/−^‐cell lines. (A) Exemplary western blots detecting amidophosphoribosyl transferase (GPAT), ferritin heavy chain (Ft, the band at 21 kDa was analyzed), and transferrin receptor (TfR). (B–E) Densiometric quantification of the western blots (*n* = 3). (F) Activity of aconitase in cytosolic fractions of the cell lines (*n* = 4). (G, H) Activities of lactate dehydrogenase (cytsolic enzyme) and citrate synthase (mitochondrial extract) as control for the fractionation (*n* = 4). Unpaired two‐tailed *t*‐test (*P < 0.05, **P < 0.01, ***P < 0.001, G–H were not analyzed). The error bars depict the standard deviations.

Gene silencing of Grx3 by siRNA resulted in an iron‐starvation phenotype. We thus analyzed the two knock‐out cell lines for Ft, GPAT, Ft, TfR, and IRP2 levels to assess the effects on iron homeostasis. In addition, we analyzed the levels of potential interaction partners of Grx3, *that is*, BolA2, Ciapin1 (anamorsin), and Ciao3. As depicted in Fig. 2, both knockout cell lines yielded similar results confirming the iron‐starvation phenotype. The levels of GPAT were reduced to approx. 35%, indicating malfunctioning FeS insertion (Fig. 2A,B). The levels of Ft were reduced to approx. 50%, and the levels of TfR increased to approx. 220% compared to the parental HeLa cells (Fig. 2A,C,D). These indicated activation of IRP binding to the respective IREs and thus functional iron‐starvation despite sufficient iron and transferrin availability in the growth medium. The activity of aconitase (holo‐IRP1) in cytosolic fractions of the cell extracts was reduced below 50%, see Fig. 2F–H, including fractionation controls, confirming the activation of IRP1. As described for the gene‐silencing study before [15], the levels of IRP2 were not affected in the knock‐out cell lines (Fig. 2A,E).

**Fig. 3:**
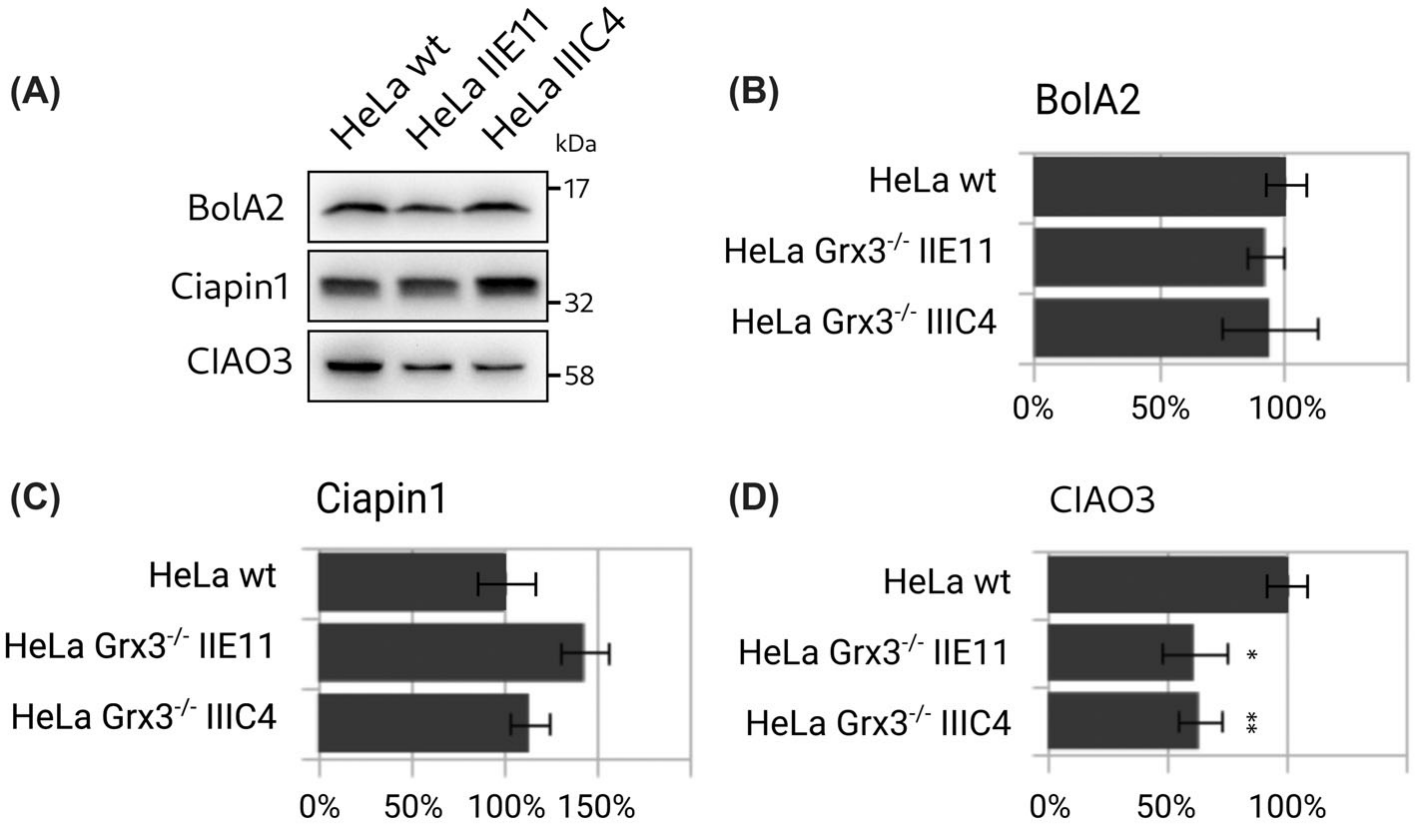
Levels of potential interaction partners of Grx3 in the Grx3^−/−^‐cell lines. (A) Exemplary western blots detecting BolA‐like protein 2 (BolA2), Ciapin1 (anamorsin), and cytosolic iron–sulfur protein assembly protein Ciao3. (B–D) Densiometric quantification of the western blots (*n* = 3). Unpaired two‐tailed *t*‐test (*P < 0.05, **P < 0.01, ***P < 0.001). The error bars depict the standard deviations.

The levels of the reported interaction partners of Grx3, *that is*, BolA2 and Ciapin1, did not significantly change due to the knockout (Fig. 3A–C). Hence, these were not upregulated to compensate for the loss of Grx3. The levels of Ciao3 were consistently reduced to approx. 60% (Fig. 3A,D).

### Rescue of the iron‐starvation phenotype with Grx3 domain constructs

**Fig. 4:**
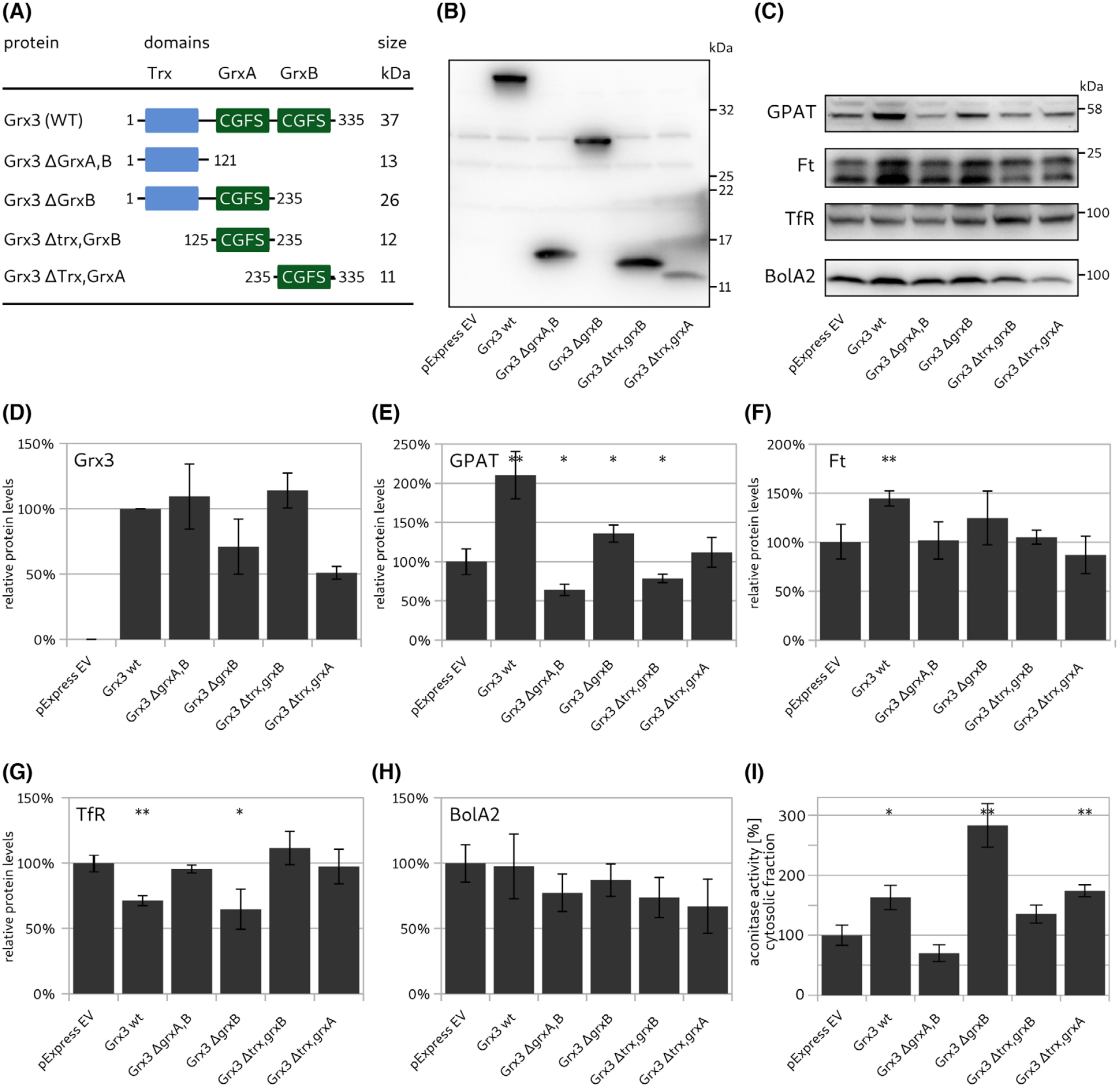
Rescue of the iron starvation phenotype by Grx3 domains and combinations thereof. (A) Construction of the domain constructs. (B) Western blot of the HeLa Grx3^−/−^‐3‐C4 cell line expressing the different Grx3 constructs depicted in A. (C) Exemplary western blots detecting amidophosphoribosyl transferase (GPAT), ferritin heavy chain (Ft, the band at 21 kDa was analyzed), transferrin receptor (TfR), and BolA‐like protein 2 (BolA2). (D–H) Densiometric quantification of the western blots (*n* = 3). (I) Activity of aconitase in cytosolic fractions of the cell lines (*n* = 4). Unpaired two‐tailed *t*‐test (*P < 0.05, **P < 0.01, ***P < 0.001, D was not analyzed). The error bars depict the standard deviations.

We generated constructs encoding the individual Grx3 domains (Trx, GrxA, and GrxB) and combinations thereof (Fig. 4A) in the expression vector pExpress under the control of a β‐actin promoter. Transfection of the KO cell line 3‐C4 with these constructs yielded protein bands of the expected sizes (Fig. 4B). The faint band at approximately 30 kDa visible in all lanes is an unspecific artifact. It is not a truncated form of Grx3; the calculated size of the longest possible product from the disrupted gene is below 13 kDa. The levels of the (re‐)introduced proteins appeared to be similar for Grx3 wild‐type, Grx3 ΔGrxA,GrxB, and Grx3 ΔTrx,GrxB, but lower for Grx3 ΔGrxB and Grx3 ΔTrx,GrxA (Fig. 4B,D). Of note, our original polyclonal antibody raised against human Grx3 did not detect the Grx3 ∆Trx,GrxB construct, while our antibody raised against mouse Grx3 detected all human protein domains. We thus cannot ensure that this approach yields equal quantitative staining for all individual domains (Fig. 4B).

The transfected 3‐C4 cells were analyzed for the impact of these domain constructs on the iron starvation phenotype compared to empty vector controls (Fig. 4). Expression of the full‐length Grx3 rescued the phenotype, *that is*, the levels of GPAT increased more than twofold (Fig. 4C,E), thus reversing the decrease of GPAT induced by the knockout of *GLRX3* (Fig. 2A,B). The levels of Ft increased, and the levels of TfR decreased (Fig. 4C,F,G), also reverting the phenotype of the *GLRX3* knock‐out (Fig. 2A,C,D). Cytosolic aconitase activity increased to 165% (Fig. 4I), indicating decreased levels of IRP activation.

Expression of Grx3 ΔGrxA,GrxB, *that is*, the N‐terminal Trx‐like domain alone, did not rescue nor affect any aspect of the *GLRX3* knockout phenotype (Fig. 4); in fact, GPAT levels dropped even further (Fig. 4E). Extension of the Trx‐like domain by the first class II Grx domain (Grx3 ΔGrxB), however, resulted in solid effects. Similar to the full length, the levels of GPAT increased (Fig. 4C,D), Ft increased somewhat (Fig. 4C,E), and the levels of TfR decreased to levels of the parental cells before *GLRX3* knock‐out (Fig. 4C,G). The activity of aconitase increased even more than when full‐length Grx3 was re‐introduced (Fig. 4I). Expression of Grx3 ΔGrxB did not change the levels of BolA2 significantly (Fig. 4C,H). The reintroduction of either Grx domain of Grx3 (ΔTrx,GrxA or ΔTrx,GrxB) did not rescue the functional iron‐starvation phenotype. Neither the levels of GPAT nor Ft increased, and TfR levels remained higher compared to the control (Fig. 4C,E–G). For the GrxA domain alone, the levels of GPAT dropped even further (Fig. 4E). Somewhat surprisingly, however, the cytosolic aconitase activity, *that is*, holo‐IRP1, increased following the introduction of the GrxB domain into the *GLRX3* knockout cells (Fig. 4I).

**Fig. 5:**
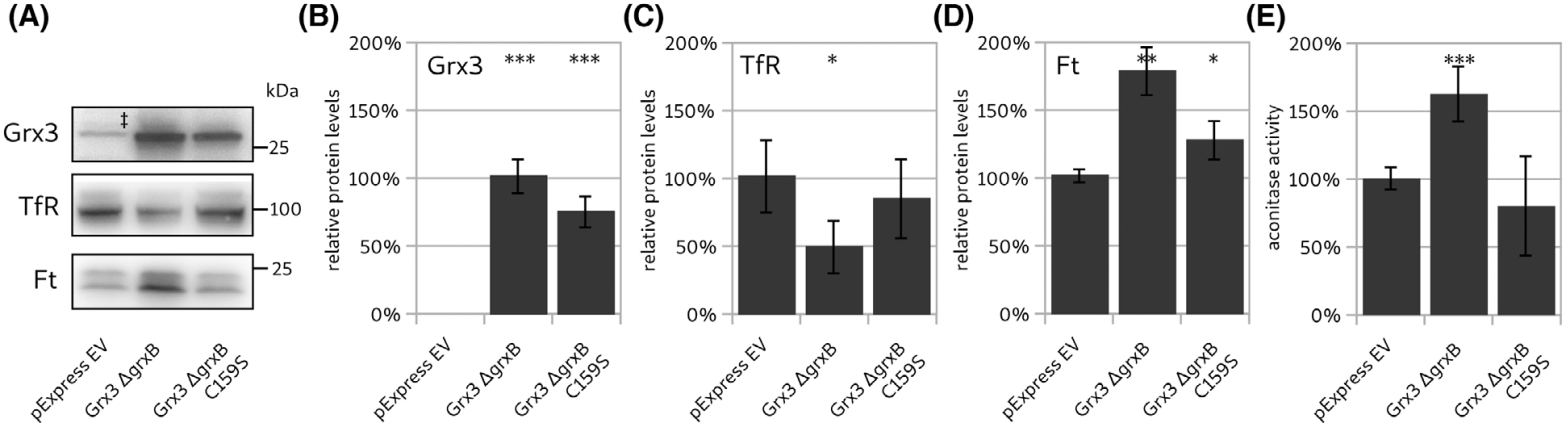
The cysteinyl residue in the active site is required for the rescue of the iron‐starvation phenotype. (A) Exemplary western blots of the HeLa Grx3^−/−^‐3‐C4 cell line expressing the ΔGrxB and ΔGrxB‐C159S constructs compared to controls transfected with empty plasmid. Grx3, ferritin heavy chain (Ft, the band at 21 kDa was analyzed), and transferrin receptor (TfR). (‡) The band visible in the knock out cell line for GrxB is the same unspecific band as discussed for the blot visible in Fig. 4B. (B–D) quantification of the western blots (*n* = 3) normalized to the knock out cell line HeLa Grx3^−/−^‐3‐C4. (E) Aconitase activity in cytosolic fractions of the transfected knock out cell line. Unpaired two‐tailed *t*‐test (*P < 0.05, **P < 0.01, ***P < 0.001). The error bars depict the standard deviations.

The requirement of the active site CGFS cysteinyl residue, and thus FeS cluster binding, for the rescue of the knockout phenotype was tested by comparing the expression of Grx3 ΔGrxB and Grx3 ΔGrxB‐C159S constructs (Fig. 5). As outlined above, expression of Grx3 ΔGrxB rescued the iron starvation phenotype, *that is*, the levels of TfR decreased (Fig. 5A,C), the levels of Ft (Fig. 5A,D), and aconitase activity (Fig. 5E) increased. In contrast, the C159S mutant of Grx3 ΔGrxB did not affect the iron starvation phenotype (Fig. 5A,C,E).

## Discussion

The homozygous disruption of the *GLRX3* gene in mice is embryonically lethal between E12.5 and E14.5 [13]. Both isogenic homozygous HeLa Grx3^−/−^‐cell lines generated here were viable and grew under normal growth conditions, *that is*, 21% oxygen, 5% CO_2_, growth medium with 10% FCS and 1 g·L^−1^ glucose. The cells grew notably slower; the generation time was approximately 2 days compared to one day for the parental HeLa cells. The morphologies of the cell lines, however, were indistinguishable (Fig. 1E–G).

The Grx family is divided into three major classes [5, 22, 29]. Class I Grxs (CPYC consensus active site) are ubiquitous glutathione‐dependent oxidoreductases; the also ubiquitous class II Grxs (CGFS active site) function in iron metabolism, while the plant‐specific class III Grxs (CC‐type) in the regulation of development. The major distinctive features between the classes are the active site motifs and the immediately preceding loop structures. These loop structures determine, together with other minor differences that relate to the binding of glutathione, the activities of class I and class II Grxs [30, 31].

Class II Grxs are critical for iron–sulfur cluster biogenesis, iron trafficking, and the regulation of iron homeostasis; for overviews, see, for instance [32, 33]. Bacteria and mitochondria usually contain a single domain class II Grx that transfers freshly synthesized Fe_2_S_2_‐clusters from the primary scaffold to target proteins [34, 35]. In addition, eukaryotic cells contain a cytosolic/nuclear multidomain class II Grx that consists of a redox‐inactive Trx‐like N‐terminal domain followed by one (yeast), two (mammals) or three (plants) class II Grx domains. The molecular functions of these proteins in iron metabolism are less well‐defined than those of their mitochondrial cousins. This study confirms the previously described loss‐of‐function phenotype [15]. Both gene silencing and knockout of the *GLRX3* gene impaired iron homeostasis and the maturation of cytosolic Fe/S‐proteins [15]. GPAT, whose levels were reduced, is translated into an inactive proenzyme. Its maturation depends on the cleavage of the pro‐peptide and the incorporation of a Fe_4_S_4_‐cluster. If this cluster insertion fails, the proenzyme is degraded. Therefore, GPAT protein levels indicate the successful maturation of FeS‐proteins [19, 20]. The protein levels of Ft, as well as cytosolic aconitase activity, were decreased. In contrast, the levels of TfR increased, which is indicative of IRP activation due to defects in iron delivery and FeS insertion. This phenotype resembles iron starvation, suggesting an inefficient utilization of the iron available at sufficient levels in the culture medium [15, 21]. We tested the ability of the individual Grx3 domains to rescue the iron starvation phenotype of the knockout cells (Fig. 4). Only the expression of either Grx3 wt or Grx3 ∆grxB with one Grx domain rescued the phenotype. Ft and GPAT levels increased, and TfR levels decreased compared to the empty vector control cells. In contrast, neither the Trx, GrxA, nor GrxB domain alone could rescue the phenotype. No significant changes in the levels of GPAT, Ft, or TfR were observed for the cells expressing these proteins (Fig. 4). Although the expression of either class II Grx domain increased the levels of active cytosolic aconitase.

BolA‐like proteins from all orders of life have been implied in iron metabolism and as potential interaction partners of class II Grxs [36]. The Grx3‐BolA2 complex was suggested to function as an Fe_2_S_2_ insertion/chaperone complex [4]. Applying immunoprecipitation and live cell proximity analyses, Frey *et al*. reported that the levels of Grx3‐BolA2 complexes increased six‐ to eightfold in response to an increase in cellular iron. The formation of heterocomplexes did not depend on the cytosolic FeS assembly (CIA) machinery, *for example*, Ciapin1 or Ciao1; instead, the Grx3‐BolA2 complexes facilitated FeS incorporation into Ciapin1, an early member of the CIA pathway [4]. The iron chaperone poly(rC)‐binding protein (PCBP) [37] can also form a heterocomplex with BolA2, bridged by glutathione and iron. It was suggested that this PCBP1‐Fe‐GSH‐BolA2 complex is required to assemble the Fe_2_S_2_ clusters on the Grx3‐BolA3 complex [38]. *In vitro*, human Grx3 can form heterodimeric and ‐trimeric complexes with BolA2. These complexes form through Fe_2_S_2_ clusters bridging between one BolA2 molecule and either the glutathione‐Grx domain [39, 40]. *In vitro*, the two FeS clusters are transferred to other proteins, such as Ciapin1 [40]. Yeast Grx3 and 4, with their single Grx domains, also form heterodimeric complexes with the BolA‐like Fra2; this complex functions as an iron sensor for iron metabolism regulation in yeast [41]. Human Grx3 can also transfer its FeS clusters directly to Ciapin1. Similar to what we report here, the transfer *in vitro* required the Trx domain and the GrxA domain, while the second GrxB domain was not strictly required. Without the Trx domain, cluster transfer from the GrxA‐GrxB domains only did not occur [42]. In our Grx3^–/–^‐cell lines, we did not observe a compensatory increase or decrease in the levels of BolA2 or Ciapin1 (Fig. 3). However, we observed a drop in Ciao3 levels to approximately 60%. This downregulation of Ciao3 could be a regulatory mechanism to decrease the FeS cluster synthesis in the cell, as it is required for the maturation of cytosolic aconitase (holo‐IRP1) [43]. This decrease in Ciao3 may also cause the lowered levels of GPAT observed here.

Grx3 has been described as a cytosolic and nuclear protein. Pham *et al*. recently analyzed the individual Grx3 domains' role in the protein's nuclear localization by fluorescence tagging and fractionation assays [44]. Under normal growth conditions, Grx3 wt was predominantly localized in the cytoplasm; treatment with diamide induced the translocation into the nucleus. Surprisingly, neither the N‐terminal Trx‐like domain nor the two class II Grx domains were required for this nuclear translocation [44]. The evolutionary advantages of the second Grx domain in human Grx3, compared to, for example, yeast, are an open question. We cannot rule out that the overexpression levels of the Trx‐GrxA construct contribute to the complementation of the iron phenotype displayed by the knock‐out cell line. The second Grx domain may be required to increase the efficiency of the protein.

In synopsis, our study demonstrates that, in cells, the minimum requirement for a functional Grx3 is the Trx domain followed by at least one class II Grx domain. Trx domains have been discussed as protein interaction modules harboring chaperone activity independent of any redox activity [45]. The domain is required to allow the Grx domain to transfer clusters efficiently to some proteins. The individual Grx domains may be able to take over some of these functions, for example, cluster supporting cluster insertion in IRP1 (Fig. 4I). However, the additional Trx domain is needed to rescue the complete phenotype of *GLRX* ^*3*−/−^‐cells. The evolutionary advantages of additional C‐terminal Grx domains in vertebrates and plants have yet to be discovered.

## Conflict of interest

The authors declare no conflict of interest.

## Author contributions

LMJ, MG, FZ, and SB performed the experiments and analyzed the data. CHL analyzed the data, designed, and wrote the study.

## Peer review

The peer review history for this article is available at https://www.webofscience.com/api/gateway/wos/peer‐review/10.1002/1873‐3468.70072.

## Data Availability

The data that support the findings of this study are available in Figs 1, 2, 3, 4, 5 and/or the supplementary material of this article. The HeLa *GLRX3* knockout cell line generated in this study is available from the corresponding author [horst@lillig.de] upon reasonable request.
